# Elementary students’ perceptions of 3Dmetric: A cross-sectional study

**DOI:** 10.1016/j.heliyon.2020.e04052

**Published:** 2020-06-11

**Authors:** Mohammad Faizal Amir, Niko Fediyanto, Hendra Erik Rudyanto, Dian Septi Nur Afifah, Hasan Said Tortop

**Affiliations:** a Elementary School Teacher Education Department, Faculty of Psychology and Educational Sciences, Universitas Muhammadiyah Sidoarjo, Sidoarjo, Indonesia; b Management Department, Faculty of Law Business and Social Sciences, Universitas Muhammadiyah Sidoarjo, Sidoarjo, Indonesia; c Elementary School Teacher Education Department, Faculty of Teacher Training and Education, Universitas PGRI Madiun, Madiun, Indonesia; d Mathematic Education Department, STKIP PGRI Tulungagung, Tulungagung, Indonesia; e Child Development Department, Faculty of Health Sciences, İstanbul Esenyurt University, Istanbul, Turkey

**Keywords:** Education, Elementary students perception, Augmented reality, Spatial ability, Mathematics education

## Abstract

Rapid changes in the 21st century demand the use of technology in learning geometry in elementary schools. One such technology is augmented reality (AR). 3Dmetric (3D and Geometric) is a geometry learning medium on AR-based 3D space material. Students' perceptions, which refer to their interpretation, are a key factor in studying the changes in their interpretations of a particular phenomenon. The purpose of the current study was to investigate the perceptions of elementary school students after using 3Dmetric to learn geometric shapes. The differences and the relationship between the students’ level of perception and level of spatial ability were also investigated. This study applied a cross-sectional approach with quantitative and qualitative designs. A total of 36 students in one elementary school in Indonesia participated in this study. The instruments used were the Perception Scale for Using 3Dmetric in Geometry Teaching, Spatial Ability Scale, and In-Depth Interview Form. Results showed that the positive perception of elementary school students regarding the use of 3Dmetric does not depend on the level of their spatial ability. Moreover, the difference in their perceptions is not caused by the level of their spatial ability. The positive findings in this cross-sectional study can contribute to the success of AR-based learning and teaching in the 21st century, especially with regard to learning materials for 3D geometry. They can also lead to the formation of the spatial abilities and improvement in the academic performance of elementary school students.

## 1 Introduction

The rapid changes in educational technology in the 21st century have influenced the use of technology in students' mathematics learning (Kay, 2010; McCulloch et al., 2018; Young, 2017). Hence, mathematics learning by researchers and educators needs to be reformed so that students can continue acquiring knowledge and skills that are appropriate to these changes. Through their language and actions, teachers can create an atmosphere in which they closely interact with their students with the help of technology. Thus, the use of technology in mathematics learning can attract students' attention toward the mathematics content (Hardman, 2019; Yin, 2016). The use of augmented reality (AR) in teaching is one example of technology that is suitable in learning to increase students’ readiness to understand learning materials.

AR is defined as “a technology that combines two-dimensional and or three-dimensional virtual objects into a real environment which is then projected in real time” (Azuma, 1997). In the context of education, AR helps students in the process of in-depth learning by connecting virtual dimensions to real dimensions (Klopfer and Sheldon, 2010; Oranç and Küntay, 2019). Therefore, AR is recommended in the learning process because it can facilitate observation that is not easily done by the naked eye (Wu et al., 2013). Although many studies report the benefits of using AR in learning, problems with usage and technical issues remain and continue to affect student performance (Akçayır and Akçayır, 2017). In addition, instead of having a high effect on learning outcomes, AR has been found to exert a moderate effect (Garzón and Acevedo, 2019); therefore, further investigation into AR needs to be conducted to increase students’ performance in the learning process.

AR integration in learning has been identified as a key factor in developing students' knowledge construction in learning geometry (Banu, 2012; Ibáñez et al., 2020). Moreover, students who successfully apply AR can be categorized as students who have mastered the basic abilities relative to realistic mathematics and geometry (Kaufmann and Schmalstieg, 2003; Piekarski and Thomas, 2003). Previous studies have shown that AR integration has implications in improving students' performance and level of spatial ability (Deshpande and Kim, 2018; González, 2015; Kaufmann and Schmalstieg, 2003; Kwiatek et al., 2019). Spatial ability enables students to visualize a geometrical object from a different point of view, rotation, and integration or from the integration of the given object's parts (Chao and Liu, 2017; Nagy-Kondor, 2014; Neubauer et al., 2018; Olkun, 2003; Sorby, 1999). Previous studies have also shown that the research on AR should further review the level of spatial ability of students.

In Indonesia, several schools have implemented AR in education as a learning medium to help students understand the concept of geometry. However, research involving AR in learning is still limited. Cahyono et al. (2018) found that the application of AR facilitates students’ understanding of 2D and 3D shapes. Lainufar et al. (2020) explained that the use of AR optimizes the quality of teaching and learning of geometry subjects. AR design can be modified for learning at the local level so that students can grasp 2D concepts on the basis of local knowledge (Widiaty et al., 2016).

Extensive studies at various levels have assessed perceptions toward AR in learning. In early childhood, AR helps boost the attraction of young children toward learning how to draw (Oranç and Küntay, 2019). At the elementary school level, the evaluation of AR modification as a mobile game reveals positive results for learning perceptions, such as growing enthusiasm, enjoyment, and curiosity (López-Faican and Jaen, 2020). Meanwhile, Majid et al., 2015 showed that the use of attractive AR by senior high school students improves their perception during learning. Di Serio et al., 2013 found that AR could increase the learning perception of middle school students and ignite students’ moods during learning activities (Ibáñez et al., 2014). In higher education, AR implementation results in a positive perception and thereby increases the quality of the learning–teaching process (Gupta and Bajaj, 2018).

Perception is identified as one's understanding or comprehension of a phenomenon being captured by their mental impression (Simpson and Weiner, 1989). One way to study the process of changing students' attitudes is to identify the differences in their self-perception (Holland et al., 2002). Therefore, perception can be regarded as a person's interpretation of a phenomenon. In the present study, perception is viewed as a student's interpretation after learning using 3Dmetric (3D and Geometric).

3Dmetric is an AR-based learning medium developed to stimulate the spatial abilities of elementary school students in constructing 3D spaces in their study of geometry subjects. According to previous studies, 3D metric was developed on the basis of a research design study with attention to spatial dimensions, namely, rotation, constructive space, reconstruction, visualization, and orientation (Amir et al., 2018b). A related study reported that the implementation of 3Dmetric through a design research study results in an increase in the level of spatial ability of elementary school students (Amir et al., 2018a). However, both of these studies did not report the perceptions after using 3Dmetric, including the differences and the relationship between the level of perception and the level of spatial ability of elementary school students. The perception after the use of 3Dmetrics at the same school level should be investigated further.

Thus far, no study appears to have explored students' perceptions, the differences in and relationships of perception variables, and the levels of perception and spatial ability after using 3Dmetric or AR in a geometry subject involving 3D geometric materials at the primary school level. In fact, the study of the differences in and the relationship among perception levels is important in cross-sectional studies because it aids the development of AR teaching strategies so that they are optimized for elementary school students as they learn geometry (López-Faican and Jaen, 2020). Comprehensive research into students' perception using a cross-sectional approach requires quantitative and qualitative techniques (Swiers et al., 2017). Therefore, the current study aims to investigate students' perceptions after using 3Dmetric in their study of geometrical shapes. In addition, the differences in and the relationship between students’ levels of perception and level of spatial ability were investigated using a cross-sectional study of elementary school students in Indonesia.

## 2 Materials and methods

### 2.1 Study method

The cross-sectional approach was used in this study to investigate perceptual variables and the differences in and the relationship of students' perception levels and spatial ability levels. This method can be relied upon because each student's perception data and spatial ability are collected after the use of 3Dmetric (Creswell, 2012). This cross-sectional study covers quantitative and qualitative designs. The quantitative approach is used to investigate perceptual variables, as well as the differences in and the relationship between students' level of perception and level of spatial ability; by contrast, the qualitative design is used to investigate the in-depth perception of students after the use of 3Dmetric (Swiers et al., 2017).

### 2.2 Participants

The participants comprised 36 fourth grade students at SDN Lemahputro 1 Indonesia, East Java Province. The elementary school students who met the following inclusion criteria were chosen as the participants in this study: (1) 8–9 years old, (2) has yet to receive materials for building space, and (3) a digital literacy score of 301–400 according to Information and Communication of Technology Literacy Assessment.

As the subjects were elementary school students, ethical clearance was secured as follows: (1) the SDN Lemahputro 1 school committee comprehensively reviewed the purpose of the questionnaire; (2) the SDN Lemahputro 1 school committee had the right to refuse the distribution of the questionnaire without providing a reason; (3) for the interviews and picture taking with the students, their guardian or parents comprehensively reviewed the purpose of the interview and observations; (4) the SDN Lemahputro 1 school committee and the guardians or parents offered their permission to use the information obtained by the study in various forms, including reports, publications, and presentations. Ethical clearance for this study was obtained from the SDN Lemahputro 1 school committee and the students’ guardians or parents.

### 2.3 Data collection tools

#### 2.3.1 Perception Scale for Using 3Dmetric in Geometry Teaching

The Perception Scale for Using 3Dmetric in Geometry Teaching (PSUDGT) is a questionnaire aimed at determining elementary school students' perceptions of using 3Dmetric in mathematics teaching. The PSUDGT was adapted from a perception questionnaire that included kinesthetic categories, media users, learning motivation, and authenticity (Cascales-Martínez et al., 2017). In this study, the PSUDGT had 12 statement items (Table 1) and applied a Likert-type scale with the choices of *strongly agree*, *agree*, *disagree*, and *strongly disagree*. The measurement of students' perceptions in using 3Dmetric offers four advantages: (1) kinesthetic, measuring kinesthetic learning style measures; (2) media users, measuring students’ understanding of 3D objects from a variety of perspectives; (3) learning motivation, measuring student involvement or activeness; (4) authenticity, measuring the contextual involvement of AR scenes in real objects (Cuendet et al., 2013).

**Table 1:** Categories and statements about perceptions in PSUDGT.

Categories of Perception	Statements
Kinesthetic	1. I can receive and process materials with 3Dmetric. 2. Only by rotating the power picture could I see the rotation of an object. 3. I became focused on learning with the activity of operating augmented reality media.
Media Users	1. For me, the use of 3Dmetric is very easy. 2. I can see 3Dmetric from various points of view. 3. Augmented reality media makes me understand more about space building materials.
Learning Motivation	1. I am more enthusiastic about learning using 3Dmetric. 2. I am more interested in learning mathematics because it uses 3Dmetric. 3. I want to use 3Dmetric for further learning.
Authenticity	1. I feel that learning is real. 2. Learning activities with 3Dmetric relate to real-life situations. 3. I feel that 3Dmetric is real.

Before being used, the PSUDGT was reviewed by experts through an item pool. This item pool is presented to experts in the fields of math education and technology education to ensure the validity of the scale. After several revisions from the experts, the PSUDGT was distributed to the students with experience in using AR in learning mathematics. The validation value of this perception was calculated in terms of the Cronbach's alpha coefficient, which was calculated using SPSS. The resulting coefficient value of 0.817 indicated the high level of internal consistency of the items in the questionnaire.

The students' level of perceptions of 3Dmetric was divided into two, namely, good perceptions and bad perceptions. Perceptions were categorized as good if the students' assessment scores fell within the range of 3–4 in the PSUDGT. Perceptions were categorized as bad if the students’ assessment scores fell within the range of 1–2 in the PSUDGT.

#### 2.3.2 Spatial Ability Scale

The Spatial Ability Scale (SAS) was proposed by Kozhevnikov et al. (2007) and includes five questions related to spatial components, namely, representation, visualization, rotation, reconstruction, and constructive space. Each item contains five subquestions; hence, the SAS comprises 25 items scored in the range of 1–5. The range of students' spatial ability was determined from the results of spatial ability tests that have been tested for validity with the results of independent sample t-tests using SPSS version 19 for Windows (Pallant, 2011). For the reliability of this scale, a Cronbach's alpha coefficient of 0.763 was calculated, and it indicated the high level of internal consistency of the items.

The level of spatial ability was divided into two, namely, low and high. Students were considered as having low spatial ability if their SAS scores for each component of spatial ability fell in the range of 0–12. Hence, the total SAS scores for the five components of spatial ability ranged from 0 to 60. Students were considered as having high spatial ability if their SAS scores for each component of spatial ability ranged from 13 to 25; their total SAS scores for the five components of spatial ability thus ranged from 61 to 125.

#### 2.3.3 In-depth Interview Form

The In-depth Interview Form consisted of nine core questions aimed at investigating students’ in-depth perception about the use of AR in mathematics learning. The interview questions were reviewed by three mathematics educators. Then, the questions were tested in an experiment with two students and revised according to the results. An example is, “What do you feel about augmented reality when you used it in mathematics learning?” Thus, a study was carried out to determine the validity of the data collection tool.

The interview protocol was implemented with the following steps: (1) the researcher chose five students to be interviewed; (2) the students selected had high and low spatial abilities; (3) the interviews were conducted outside school hours; (4) the interviews were conducted after the students received the whole set of 3Dmetric learning; (5) the interviews were conducted one on one and were semistructured with reference to the categories of perception in Table 2; (6) the data obtained in the interviews were transcribed.

**Table 2:** Four themes and 12 codes of perception.

Themes	Codes
Kinesthetic	Receive Rotate picture Activity in operation
Media Users	Easy to use 3D space from various angles Understand 3D space
Learning Motivation	Enthusiastic in learning Interested in geometry Next learning
Authenticity	Learn by reality Correlated Real media

### 2.4 Data collection procedures

This study used a data collection procedure in stages. The distribution of the research instrument to the 36 students took one month. The research procedure for investigating students' perceptions toward the use of AR was divided into four stages: (1) students' spatial abilities were measured first and then grouped into high and low using the SAS; (2) the students were given learning trajectory (LT) in the form of the use of 3Dmetric in building materials under the supervision of their teachers; (3) the PSUDGT was given after the LT to measure the students’ perceptions of using 3Dmetric; (4) in-depth interviews were then conducted.

### 2.5 Data analysis

The data in this study consisted of quantitative and qualitative data. Data analysis was carried out independently, and the results were discussed and modified until a consensus among researchers was reached (Fawaz and Anshasi, 2019; Gerritsen-van Leeuwenkamp et al., 2019). The interrater reliability score between researchers was 8.75 (good).

The quantitative data were subjected to a normality test, paired t-test, and prevalence ratio test. The information collected from the tests was converted into a spreadsheet on SPSS. The normality test is performed to determine whether the distribution of data is normal or not (Montague and van Garderen, 2003). Data normality test in the form of the Shapiro–Wilk test is used if the sample size is ≤ 50; the result is transformed into *p* and assumed to be normal (Royston, 1992). The data obtained from PSUDGT had a Sig. of 0.765, whereas the data from the SAS had a Sig. of 0.603. As the values of Sig. for the two datasets >0.05 on the basis of the normality test, then the students' perception data and spatial ability data were normally distributed. The differences between the perception variables and between the level of perception and the level of spatial ability were tested using paired t-tests. The level of statistical significance (p) was set to 0.05. To adjust for possible interactions and confounding factors, the researchers conducted a regression analysis to determine the students' perceptions. Meanwhile, the relationship between the students’ level of perception and their level of spatial ability was explained in terms of the prevalence ratio (PR) with 95% confidence interval (Setia, 2016).

The qualitative data were subjected to descriptive content analysis, which describes the iterative process of mapping messy data to the most critical themes obtained from interview transcripts, field notes, and class observation recordings. Conventional content analysis is generally used with study designs that aim to describe phenomena, such as students’ perception of 3Dmetric in geometry learning in the current work. This type of design is usually appropriate when existing theories or research on a phenomenon is limited. The process contains six steps: familiarization with data, assignment of preliminary codes to describe the content of data, search for patterns or themes in systems across different interviews, review of themes, definition and naming of themes, and report production (Miles et al., 2014).

The quantitative data and qualitative data of perception were coded manually to simplify the analysis and presentation of data in the current work. The coding of quantitative data was carried out by providing consecutive codes A, B, C, and D for the categories of perception and numbering them accordingly. For example, the kinesthetic categories for statements 1, 2, and 3 were A1, A2, and A3, respectively. The coding of the qualitative data was carried out by content analysis, that is, creating themes and perceptual data analysis codes (Table 2) associated with citations or incidents that are representative of the research topic.

Two researchers took part in the content analysis. The percentages of compliance of the researchers in terms of creating codes and themes showed a high agreement. Such coordination is important in ensuring the reliability of the data collection tool (Creswell, 2012).

This study also applied coding on the five students interviewed to distinguish their perceptions according to their spatial ability and age. The coding was carried out by assigning codes S1 to S5 and then adding a sex code (F for female and M for male) and age code. For example, student 1 is male and 8 years old; hence, the student is coded as S1M8.

## 3 Results

### 3.1 Use of 3Dmetric

**Figure 1:**
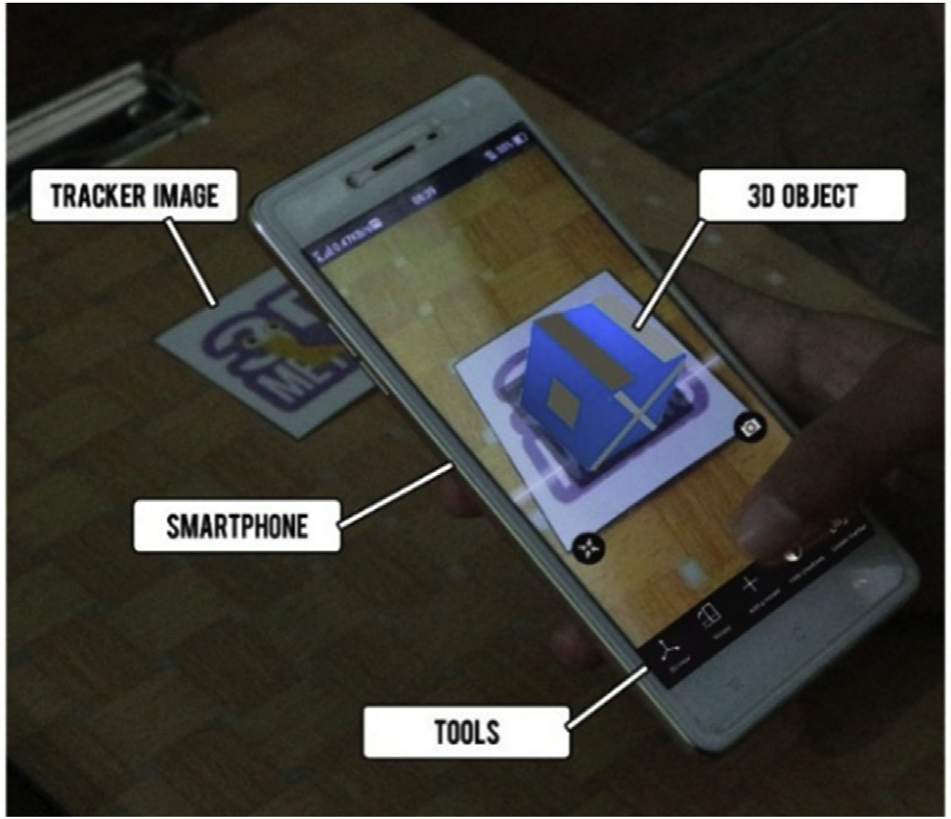
Tools in 3Dmetric.

3Dmetric has several tools, namely, image tracker, 3D models, and features such as rotation and 3D view (Figure 1). The image tracker functions as the medium detection in AR. The 3D object is a display of objects built in accordance with the components of spatial ability on mobile phones. The features of 3Dmetric help students to rotate 3D objects and create 3D views so that objects look realistic.

The process of using 3Dmetric has several stages, starting from initial design to retrospective analysis (Amir et al., 2018b). 3D models can be created using either the Blender application on a computer or laptop or Unity. For the realization of 3D models, the Augment application in Play Store can be used. Other applications include Unite AR, Vuforia, and many others. Similar to Augment, it input all the data needed through the website server of each application. In the current work, after using 3Dmetric in learning, the students provided their corresponding responses.

**Figure 2:**
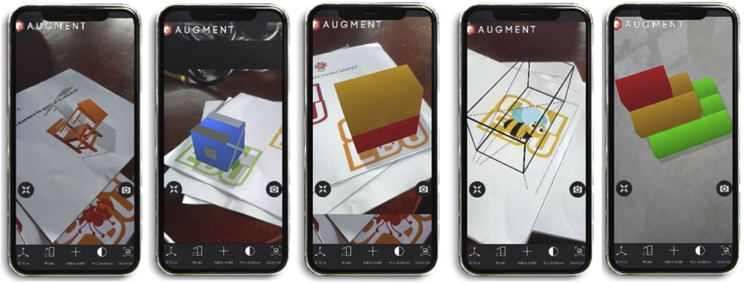
3Dmetric display on each LT based on spatial activity: (1) representation, (2) visualization, (3) rotation, (4) reconstruction, (5) constructive space.

In this research, the students’ activities in 3Dmetric followed the five steps of LT, which were designed on the basis of the spatial components: (1) representation: the students drew objects around by using geometric models; 2) visualization: the students visualized the construction of space becoming building nets; 3) rotation: the students predicted the image of the building when the building is rotated; 4) reconstruction: the students determined the position of a building space; and 5) constructive space: the students described the side view of the building space. Figure 2 shows the 3Dmetric display on each LT.

**Figure 3:**
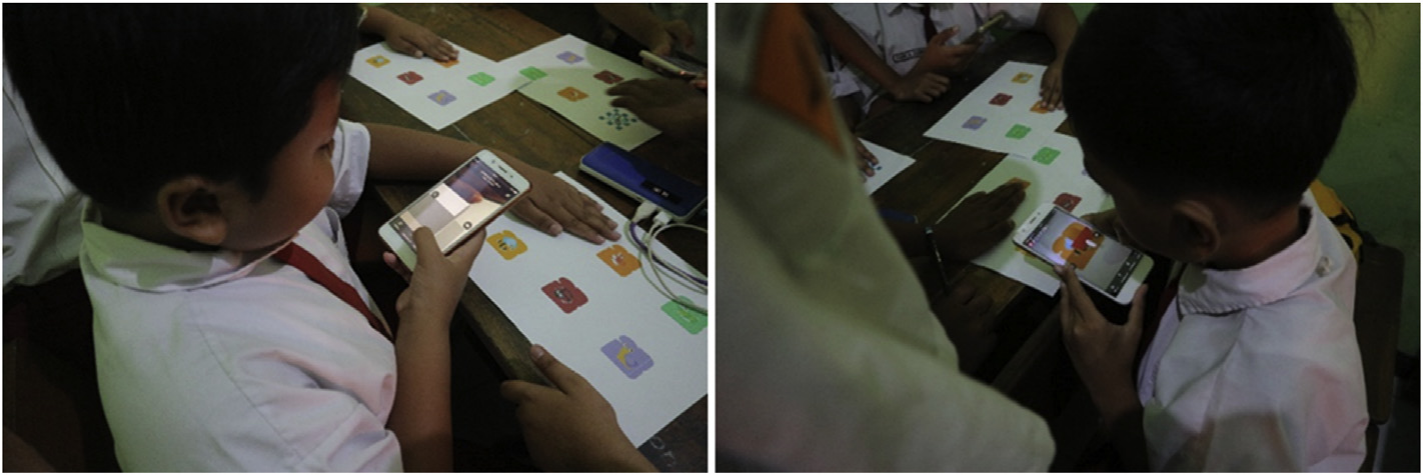
Students using 3Dmetric.

During the process of using 3Dmetric on each LT, the student activities were divided into two sessions, namely, individual sessions and group sessions. The individual sessions allowed the students to get accustomed to using 3Dmetric on their mobile phones. The group sessions allowed the students to discuss among themselves as they went through each LT step in 3Dmetric. During these activities, the students were facilitated by one companion. The students were also allowed to seek clarifications from their companion. Figure 3 shows examples of student activities in 3Dmetric.

In each LT, 3Dmetric allows students to represent the back of a building; draw a space with provisions of rotation; determine the diagonal side and diagonal part of the space; and describe the top, side, and front views of an area. The results indicated that 3Dmetric provided a realistic medium for learning 3D objects while increasing students’ spatial ability.

### 3.2 Students perception in each category

Table 3 shows the means and standard deviations of perception of the 36 students toward 3Dmetric. The mean of the student response toward 3Dmetric for all items in the PSUDGT is above 3.00, indicating that the students generally had positive perceptions toward 3Dmetric. The average value of the students’ perceptions for point B2 (3.16) was the lowest. This result indicated that when using 3Dmetric, the students referred to an explanation of the use of the medium.

**Table 3:** Means and standard deviations of students’ perception scores for the four categories.

	N	Mean	SD
**Category A**			

**A1**	36	3.61	0.49
**A2**	36	3.44	0.69
**A3**	36	3.61	0.64
	36	3.53	0.61

**Category B**			

**B1**	36	3.33	0.79
**B2**	36	3.16	0.91
**B3**	36	3.13	0.79
	36	3.21	0.83

**Category C**			

**C1**	36	3.31	0.85
**C2**	36	3.47	0.61
**C3**	36	3.33	0.89
	36	3.37	0.78

**Category D**			

**D1**	36	3.38	0.87
**D2**	36	3.25	0.91
**D3**	36	3.25	0.99
	36	3.29	0.92

The highest value of students’ perceptions is 3.61 (points A1 and A3), indicating that the use of 3Dmetric can improve the kinesthetic learning style of students with experience in spatial ability activities that require kinesthetic learning styles. These results indicate that students have an interest in using 3Dmetric and that they consider the use of 3Dmetric as easy.

In addition to the statements representing aspects of learning motivation with a mean value of 3.37, the results indicate that the use of 3Dmetric can motivate student learning. The students are enthusiastic about learning geometry using 3Dmetric, as stated in item C3 with a mean value of 3.33.

As for the authenticity of the students’ perceptions of 3Dmetric, the mean is 3.29, but the standard deviation is high at 0.92. This result indicates that the students perceive 3Dmetric as real learning through smartphones despite the absence of concrete objects or real objects.

### 3.3 Different perceptions on spatial abilities

Table 4 shows the results of the independent sample t-test using SPSS version 19 for Windows for each statement in the PSUDGT and the overall perception assessment according to the level of spatial ability in 3Dmetric. Whether those with low spatial ability hold negative perceptions cannot be ascertained. The students with low spatial ability provided a more positive perception than those with high spatial ability, particularly for items A2, A3, C2, C3, and D1. For items A1, B2, B3, C1, D2, and D3, the students with high spatial ability provided a more positive perception than those with low spatial ability. For item B1, both groups provided an average perception value of 3.00.

**Table 4:** Results of independent sample t-test.

	Students with low spatial ability (n = 15)		Students with high spatial ability (n = 21)		T	df	Sig. (two-tailed)
	M	SD	M	SD			
**A1**	3.60	0.51	3.62	0.49	-0.12	34	0,91
**A2**	3.46	0.63	3.43	0.74	-0.16	34	0.87
**A3**	3.80	0.41	3.47	0.75	1.51	34	0.14
**B1**	3.33	0.89	3.33	0.73	0.00	34	1.00
**B2**	3.00	1.06	3.28	0.78	-0.93	34	0.36
**B3**	3.13	0.74	3.14	0.85	-0.35	34	0.97
**C1**	3.26	0.79	3.33	0.91	-0.23	34	0.82
**C2**	3.6	0.51	3.38	0.67	1.06	34	0.29
**C3**	3.53	0.91	3.19	0.87	1.14	34	0.26
**D1**	3.46	0.74	3.33	0.96	0.45	34	0.66
**D2**	3.2	0.94	3.28	0.90	-0.27	34	0.78
**D3**	2.93	1.22	3.47	0.75	-1.65	34	0.11

The perceptions of the students with low and high spatial abilities did not differ. The mean scores of the two groups also showed significance. Hence, both groups had the same positive perception of 3Dmetric in each category.

### 3.4 Correlation of perception level and spatial ability level

One of the characteristics of a cross-sectional study is its use of PR in statistical calculations. In this work, the level of perception is categorized as good perception and bad perception. When PR is calculated and the ρ-value (statistical significance) is obtained, the significance value is not the same. A case is said to be asymmetrical when the p-value results are unequal. The magnitude of the difference between ρ-values depends on the difference between the proportions being compared; hence, if two proportions are close to each other (around 50%), then the difference between the two ρ-values will not be too “dramatic” (Cumminngs, 2009). Table 5 shows that the students with high spatial ability have a proportion = 53.6%, which is classified as good perception; whereas those with low spatial ability have a proportion = 51.1%. The two proportions do not greatly differ, and thus, the difference in p-values is not significant.

**Table 5:** Prevalence ratio of the correlation between students’ spatial ability and perception control.

	Good Perception			Bad Perception		
	Proportion n (%)	Prevalence Ratio (95% Cl)	ρ-value	Proportion n (%)	Prevalence Ratio (95% Cl)	ρ-value
High Spatial Ability	135 (53.6)	1.48 (1.15–1.90)	0.003	117 (46.4)	0.56 (0.33–0.96)	0.04
Low Spatial Ability	92 (51.1)	1.23 (1.05–1.45)	0.01	88 (48.9)	0.79 (0.65–0.95)	0.01
Prevalence Ratio = 1.015						

In a cross-sectional study, researchers measure student outcomes and exposure at the same time. Researchers can study the relationship between these variables. They can also recruit study participants and examine the results in this population or estimate the prevalence of results in those surveyed. Table 5 shows the homework value of 1.015, which means that the level of spatial ability does not affect the level of perception about the use of 3Dmetric.

### 3.5 Obtaining an in-depth understanding of perception

Interview data use several factors that contribute to teachers’ development or assessment of complex perceptions (Clarke et al., 2013; Grierson and Gallagher, 2009; Loong et al., 2017). The interview results in the current work highlight the factors that cause positive perceptions toward using 3Dmetric. Evidence from factors in each category is provided subsequently.

For category A (kinesthetic), several students showed a good focus in learning geometry through AR because the 3Dmetric helped them rotate images so that they could understand the side diagonals and the diagonal of the space on the building space. The interviews showed that the students could rotate a space just by turning the target image according to the instructions of 3Dmetric. The students said that with 3Dmetric, they were able to focus on learning the material building space. Others stated that they could understand and process materials with 3Dmetric properly. An example is shown as follows:

*...It's really exciting, sir, to excite the thrill until it's not easy to receive and process the material so it's easy. Lots of materials can be obtained from this* 3Dmetric. (S1M8, A1)

...*No, it's just a computer game, and the target image continues to rotate. To know how an object is being rotated or rotated*. (*S4F8*, A2)

...*Yes, I became focused on learning with 3Dmetric. I don't know what to focus on in learning because when I first learned it, I didn't use this medium, Like learning, it was heavy, so I didn't focus too*. (S2M9, A3)

In category B, some students had difficulty in using 3Dmetric because of their inexperience in using smartphones. Nonetheless, for some of the students, the instructions helped or alleviated their difficulties in using 3Dmetric.

...*Maybe the initial start time is still stuttering. Over time, it's easy to use. Moreover, there are clear instructions to use. So even without supervision by the teacher, you can use it yourself*. (S3F8, B1)

...*I can still use the medium without being supervised by teacher Sis. But a friend of mine can't use it and always asks me or the teacher for help. There might be reasons, like he might have never used a smartphone before*. (S4F8, B1)

In category C, some students immediately gained interest in 3Dmetric because it allowed them to play with smartphones especially equipped for AR. As they used it for the first time, their understanding of geometry was achieved easily. The students’ interest indicated increased learning motivation.

...*If you use 3Dmetric, this will make you excited about learning geometry because you are curious about the model that appears on the smartphone screen. This is the first time I'm using this medium. This is the spirit of learning using this medium. This AR medium boosted my enthusiasm in learning geometry. If possible, all subjects should use this medium*. (S2M9, a combination of C1 and C3)

...*At that time, I used it for a quiz. The lesson was more interesting using 3Dmetric because there were many materials about geometry that could be obtained*. (S4F8, C2)

In category D, some students felt that 3Dmetric is real or genuine. However, they thought that 3Dmetric is real in terms of modeling and can thus facilitate the learning process.

...*I think that I have benefited from using 3Dmetric. In my opinion, this geometry object is so real that I can't feel any boundaries between virtual and reality*. (S3F8, a combination of D1 and D2)

...*How come the model in the medium looks real? The first time I saw it, I was shocked. How come a chair could appear when there was no chair in the picture?* (S1M8, D3)

The result also showed that the students believed that the use of 3Dmetric makes learning geometry fun and less frightening. 3Dmetric trains students' spatial abilities and helps them easily understand 3D building materials, thereby leading to improvements in students’ academic performance.

## 4 Discussion and conclusion

This study provides the results of an investigation into the perceptions of the students of a primary school in Indonesia toward the use of 3Dmetric. Results are obtained from two levels of spatial abilities of students on the basis of a cross-sectional study. The main finding is that students with high and low spatial abilities have positive perceptions. This positive perception is the same as that expected from elementary students when using AR-based media (Cabero-Almenara et al., 2019; López-Faican and Jaen, 2020; Wen and Looi, 2019). That is, do not directly lead to successful learning. Although previous studies have indicated decreased cognitive load in the AR-based learning context Jahnke and Meinke Kroll (2018), these definitions may imply that learners’ perceived cognitive load is not the sole essential factor affecting their spatial ability (Amir et al., 2018a).

One of the important findings in this study is that students make a good contribution, which is reflected in their increased motivation to learn geometry in a 3D space. Qian (2014) and Chang and Hwang (2018) found that in learning visual arts, the learning motivation of attention and satisfaction are better rated in an AR-based learning environment than in a slide-based learning environment. When the students’ learning motivation in AR-based learning was independently surveyed, the attention and confidence of the students were highly rated.

In addition, positive perceptions and increased motivation arise because of the support of LT, which guides the formation of students’ spatial abilities during learning activities using 3Dmetric. Previous studies found that through spatial-based activities, particularly representation, visualization, rotation, reconstruction, and constructive space, the spatial ability of elementary school students increases. The same proposition was stated by Ovbiagbonhia et al. (2019), who explained that the spatial ability of students rises during activities using AR and will thus lead to positive student perceptions.

The results of the PR reinforce the finding that no relationship exists between perception and spatial ability. Hence, the students’ level of perception toward using 3Dmetric is not influenced by the level of their spatial ability. The same result is generated by the interviews, in which the students provided a good perception of the use of 3Dmetric in learning geometry. The researchers noted several observations when interviewing the students after using 3Dmetric to determine student responses and raise awareness of using AR media. Students can use kinesthetic learning styles when using such media. Students can learn better when they experiment or use AR media directly in learning (Medina et al., 2019; Tyas and Safitri, 2017) so that the materials can be appropriately conveyed.

Aside from growing positive student perceptions and increasing their spatial abilities, 3Dmetric boosts elementary school students' cognitive performance in 3D space geometry. The literature review and the findings of this study show that 3D images that sink in the real world are worth more than others (images or words). Alternatively, as the elementary students admitted, 3Dmetric helps them easily understand 3D space geometry because AR supports visualization and interaction. Thus, we can conclude that 3D technology, such as 3Dmetric, enhances the teaching and learning of mathematics, especially for elementary school students. In sum, the 3Dmetric application can increase information usage and access to knowledge and boost digital inclusion and information. However, other results indicate that the mean of students’ perceptions of media users is lower than the mean of other categories. Although 3D technology is still at its infancy in terms of their application in education/teaching, AR has been implemented and studied by different authors in various fields (Chen, 2019; Dominguez et al., 2012; Kamarainen et al., 2013).

In this work, we conclude that elementary school students in Indonesia have a positive perception of 3Dmetric usage regardless of the level of their spatial ability. The difference in their perceptions is not influenced by the level of their spatial ability.

### 4.1 Recommendations

The positive findings of this cross-sectional study can contribute to the success of AR-based learning and teaching in the 21st century, especially in terms of geometrical materials for learning 3D geometry. They can also lead to the formation of spatial abilities and boost the academic performance of elementary school students.

Although the participants had a positive perception toward using 3Dmetric, this work involved only one primary school in Indonesia. In addition, student activities during the process of using 3Dmetric in learning were not measured. Therefore, researchers need to be careful in generalizing the results for other elementary school students or other school levels and with regard to the relationship of perception with student activities. As a recommendation, further research should involve an investigation into the relationship of perception with student activity during the use of 3Dmetric involving a larger number of students.

## Declarations

### Author contribution statement

M.F. Amir, N. Fediyanto, H.E. Rudyanto, D.S. Nur Afifah, H.S. Tortop: Conceived and designed the experiments; Performed the experiments; Analyzed and interpreted the data; Contributed reagents, materials, analysis tools or data; Wrote the paper.

### Funding statement

This did not receive any specific grant from funding agencies in the public, commercial, or not-for-profit sectors.

### Competing interest statement

The authors declare no conflict of interest.

### Additional information

No additional information is available for this paper.
